# Bio-artificial pleura using autologous dermal fibroblast sheets to mitigate air leaks during thoracoscopic lung resection

**DOI:** 10.1038/s41536-020-00113-z

**Published:** 2021-01-04

**Authors:** Masato Kanzaki, Ryo Takagi, Kaoru Washio, Mami Kokubo, Shota Mitsuboshi, Tamami Isaka, Masayuki Yamato

**Affiliations:** 1 The Department of Thoracic Surgery, Tokyo Women’s Medical University, Tokyo, Japan; 2 Institute of Advanced Biomedical Engineering and Science, Tokyo Women’s Medical University, Tokyo, Japan

**Keywords:** Tissues, Tissue engineering

## Abstract

Lung air leaks (LALs) due to visceral pleura injury during surgery are a difficult-to-avoid complication in thoracic surgery (TS). Reliable LAL closure is an important patient management issue after TS. We demonstrated both safeties of transplantation of a cultured human autologous dermal fibroblast sheet (DFS) to LALs. From May 2016 to March 2018, five patients who underwent thoracoscopic lung resection met all the inclusion criteria. Skin biopsies were acquired from each patient to source autologous dermal cells for DFS fabrication. During the primary culture, fibroblasts migrated from the dermal tissue pieces and proliferated to form cell monolayers. These fibroblasts were subcultured to confluence. Transplantable DFSs were fabricated from these subcultured fibroblasts that were trypsinized and seeded onto temperature-responsive culture dishes. After 10 days of fabrication culture, intact patient-specific DFS were harvested. DFSs were analyzed for fibroblast cell content and tissue contaminants prior to application. For closing intraoperative LAL, mean number of transplanted autologous DFS per patient was 6 ± 2 sheets. Mean chest drainage duration was 5.0 ± 4.8 days. The two patients with major LAL had a drainage duration of more than 7 days. All patients currently have no LAL recurrence after discharge. DFSs effectively maintain LAL closure via remodeling of the deposited extracellular matrix. The use of autologous DFSs to permanently close air leaks using a patient-derived source is expected to reduce surgical complications during high-risk lung resections.

## Introduction

With continual advances in minimally invasive thoracic surgery (TS) techniques, video-assisted thoracoscopic surgery (VATS)—including robotic surgery—is increasingly used in TS^1–3^. In particular, lung wedge resection is generally a relatively simple procedure using surgical stapling devices, and most cases are now performed by VATS^4,5^. Nonetheless, lung air leaks (LALs) due to visceral pleura injury during surgery is a complication difficult to avoid in TS. Despite various precautions taken during the operation, such as staples, adhesives such as fibrin glue, pleural tenting, and fissure-less surgery, LALs occur in up to 20% of patients. Approximately 5–10% of patients who undergo TS continue to have an air leak after 5 days^6,7^. Unresolved LALs are associated with prolonged chest tube drainage and costly long-term hospitalization. LAL closure is an important patient management challenge after TS. Of the various methods used for intraoperative leak closure, fibrin-based biological tissue adhesives have been used for intraoperative air leaks for over 20 years^8–11^. However, fibrin glue does not reduce the hospitalization and chest tube drainage period in LAL cases during surgery compared to conventional methods. Similarly, other biological adhesives and artificial synthetic materials for lung repair have low biocompatibility due to low adhesion to and compatibility with living tissue.

We previously reported a cell sheet engineering technique for LAL sealants using autologous dermal fibroblast sheets (DFSs) harvested from temperature-responsive culture dishes, and the first success of intraoperative LAL closure using autologous DFS in both animal models and clinical studies^12–14^. In this study, we demonstrated the safety of transplantation of a cultured autologous DFS and the effectiveness of the DFS in closing LAL during surgery.

## Results

### Fabrication of transplantable autologous DFS

**Fig. 1:**
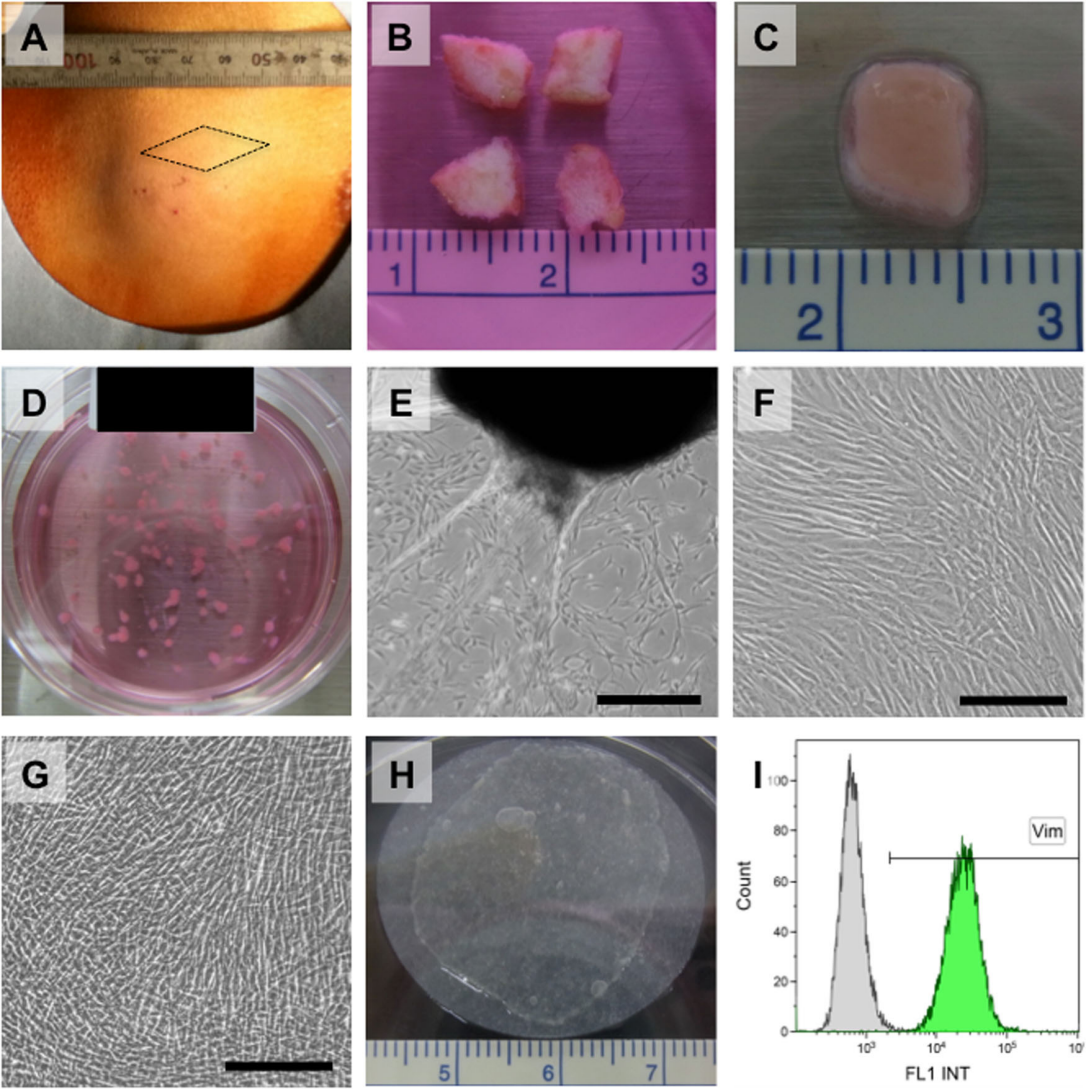
Preparation of autologous dermal fibroblast sheet. **A** Skin was excised from the planned surgical location of the skin incision from the lateral chest under local anesthesia. **B** A 5-mm square of tissue was prepared using dissecting scissors. **C** A 5-mm square of tissue underwent enzymatic treatment in a culture dish in a carbon dioxide gas incubator for 2–3 h. **D** The 1-mm square tissue pieces were transferred into a 60-mm dish for primary culture. **E** Phase-contrast micrographs of fibroblasts that migrated from transferred tissue pieces after 17 days in explant culture. Scale bar indicates 500 µm. **F** Phase-contrast micrograph of fibroblasts at 4 days after subculture on a 100-mm culture dish. Fibroblasts proliferated to form monolayers. Scale bar indicates 200 µm. **G** Phase-contrast micrograph of fibroblasts at 10 days after seeding onto temperature-responsive culture dishes. Fibroblasts proliferated and were brought to confluence. Scale bar indicates 200 µm. **H** Intact 30-mm-diameter sheet of dermal fibroblasts was harvested by simple temperature reduction to 20 °C, with the use of a support membrane (CellShifter™). **I** Both the viability of the fibroblasts and the rate of vimentin-positive cells exceeded 70%.

Timelines of fabrication of autologous DFS are shown in Table 1. Skin biopsies (~1 cm^2^) were excised from the planned surgical location of the skin incision from the lateral chest in all cases, prepared and cultured to yield patient-specific autologous fibroblasts (Fig. 1A). The skin tissues in explant cultures are shown in Fig. 1B–D. Dermal fibroblasts migrated from tissues, and cells were subcultured on 100-mm culture dishes (Fig. 1E). Results of the explant culture are shown in Table 2. Subcultured fibroblasts are shown in Fig. 1F and treated with trypsin-EDTA to seed onto 60-mm temperature-responsive culture dishes for fabricating DFS. Results of the subculture are shown in Table 3. Viable intact 30-mm diameter cell sheets comprising autologous dermal fibroblasts were harvested by simple temperature reduction to 20 °C with the use of a CellShifter support membrane (Fig. 1G, H). Autologous DFS was prepared and harvested in the clean room of the cell processing facility for patients prior to surgery, and numbers of transplanted DFS on LAL are shown in Table 4.

**Table 1:** Timeline of fabrication of dermal fibroblast sheets (DFS).

Patient number	1	2	3	4	5	Mean ± S.D.
Total fabrication period (days)	39	37	38	35	35	37 ± 2
Explant culture (days)	24	23	24	21	21	23 ± 2
Subculture (days)	5	4	4	4	4	4
Fabrication culture of DFS (days)	10	10	10	10	10	10

*S.D.* standard deviation.

**Table 2:** The results of explant culture.

Patient Number	1	2	3	4	5	Mean ± S.D.
Wet weight of dermal tissue (mg)	144	140	197	402	240	225 ± 107
No. of total cells (×10^4^ cells)	250	119	666	100	17.0	230 ± 257
Cells/skin tissue (×10^4^ cells/mg)	1.74	0.850	3.381	0.249	0.071	1.26 ± 1.35
Cell density (×10^4^ cells/cm^2^)	2.98	1.89	7.93	1.19	0.20	2.84 ± 3.02
% cell viability	97.6	96.3	96.7	99.6	98.8	97.8 ± 1.4

*S.D.* standard deviation.

**Table 3:** The results of subculture.

Patient Number	1	2	3	4	5	Mean ± S.D.
Seeding density (×10^4^ cells/cm^2^)	0.500	0.500	0.500	0.454	0.309	0.453 ± 0.083
Cell density (×10^4^ cells/cm^2^)	3.89	2.60	8.38	6.03	3.45	4.87 ± 2.33
No. of cellular division	2.96	2.38	4.07	3.73	3.48	3.32 ± 0.67
Doubling time of cells (days)	1.69	1.68	0.98	1.07	1.15	1.31 ± 0.34
%cell viability	99.5	96.0	99.9	99.4	99.6	98.9 ± 1.6

*S.D.* standard deviation.

**Table 4:** The results of fabrication of dermal fibroblast sheets.

Patient Number	1	2	3	4	5	Mean ± S.D.
Seeding density (×10^4^ cells/cm^2^)	1	1	1	1	1	1
Cell density (×10^4^ cells/cm^2^)*	11.4	10.9	21.2	12.9	9.90	13.3 ± 4.6
No. of cellular division*	3.51	3.44	4.41	3.69	3.31	3.67 ± 0.43
Doubling time of cells (days)*	2.56	2.61	2.04	2.44	2.72	2.47 ± 0.26
Total division after explant culture*	6.47	5.82	8.48	7.42	6.79	7.00 ± 1.01
% cell viability*	99.0	98.0	98.9	98.6	99.6	98.8 ± 0.6
% vimentin-positive cells*	99.6	97.3	96.4	99.3	99.5	98.4 ± 1.5
Number of transplanted the sheets	6	8	2	6	6	6 ± 2

*S.D.* standard deviation.

*The tests were carried out before 1 day of the transplantation.

One DFS per patient was validated prior to surgical transplantation. Validation standards were based on cell density, percent of cell viability (>70%), and a fraction of vimentin-positive cells (>70%) (Fig. 1I). The cell density of DFS ranged from 9.9 × 10^4^ to 21.2 × 10^4^ cells/cm^2^ (mean ± S.D.: 13.3 ± 4.6 × 10^4^ cells/cm^2^). Percent of cell viability ranged from 98.0 to 99.6% and vimentin-positive cells ranged from 97.3 to 99.6%. These validated results are shown in Table 4. Culture media collected from tissue explant culture and DFS fabrication on temperature-responsive culture dishes were cultured for bacterial and fungal contamination. LAMP and Limulus amebocyte lysate assay of culture media were also performed to detect *Mycoplasma pneumoniae* and endotoxin, respectively. No bacteria, fungi, or mycoplasma were found in any media.

**Fig. 2:**
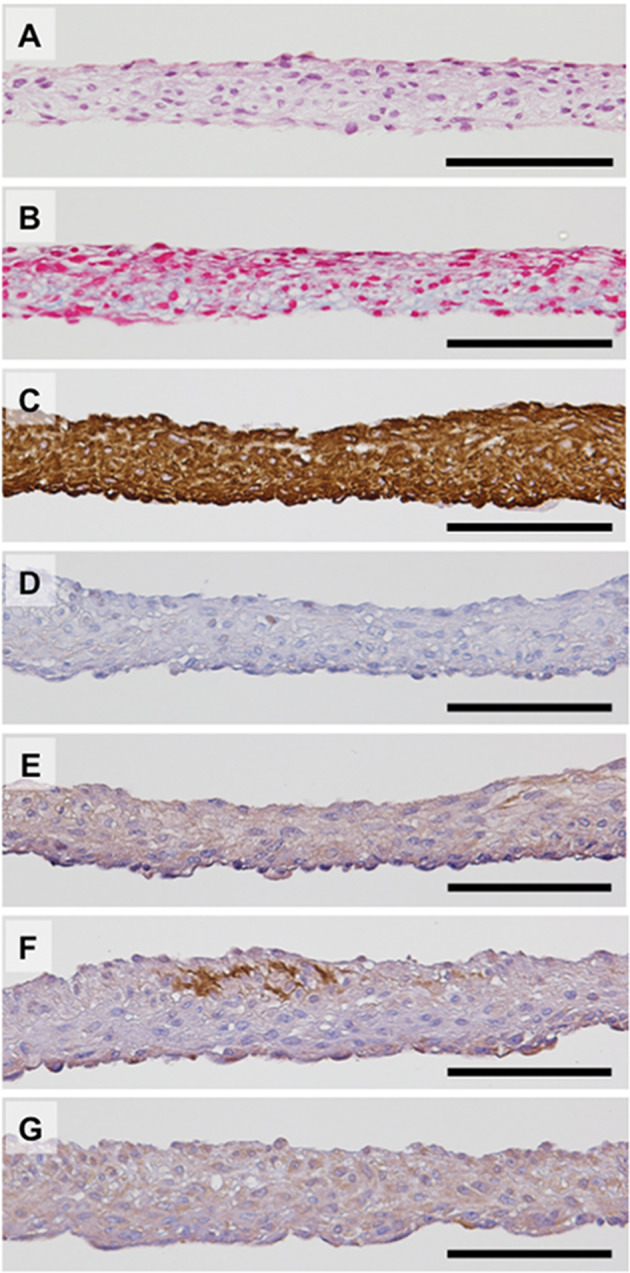
Characterization of autologous dermal fibroblast sheet. Autologous dermal fibroblast sheets (DFSs) can be harvested by low-temperature treatment and are harvested as intact sheets. Hematoxylin and eosin (**A**) and Azan (**B**) staining show that DFSs are composed of two to seven cell layers. Azan staining also showed extracellular matrix present within the harvested DFSs. Immunohistological analysis is shown for expression of vimentin (**C**), alpha-smooth muscle actin (**D**), collagen type 1 (**E**), collagen type 3 (**F**), and fibroblast growth factor 2 (**G**). Scale bars indicate 100 μm.

In two cases of examinees, DFS not transplanted on LAL were used for histological analysis (Fig. 2). Final temperature-harvested DFSs consisted of multi-stratified cell-dense layers (Fig. 2A) with relatively little extracellular matrix (ECM) present (Fig. 2B). The immunohistological analysis demonstrated that nearly all cells in DFS expressed vimentin, and some cells expressed α-SMA known as a myofibroblast marker (Fig. 2C, D). Expression of collagen type 1, collagen type 3, and FGF-2 was also confirmed (Fig. 2E–G).

### Surgical conditions and clinical outcomes

**Fig. 3:**
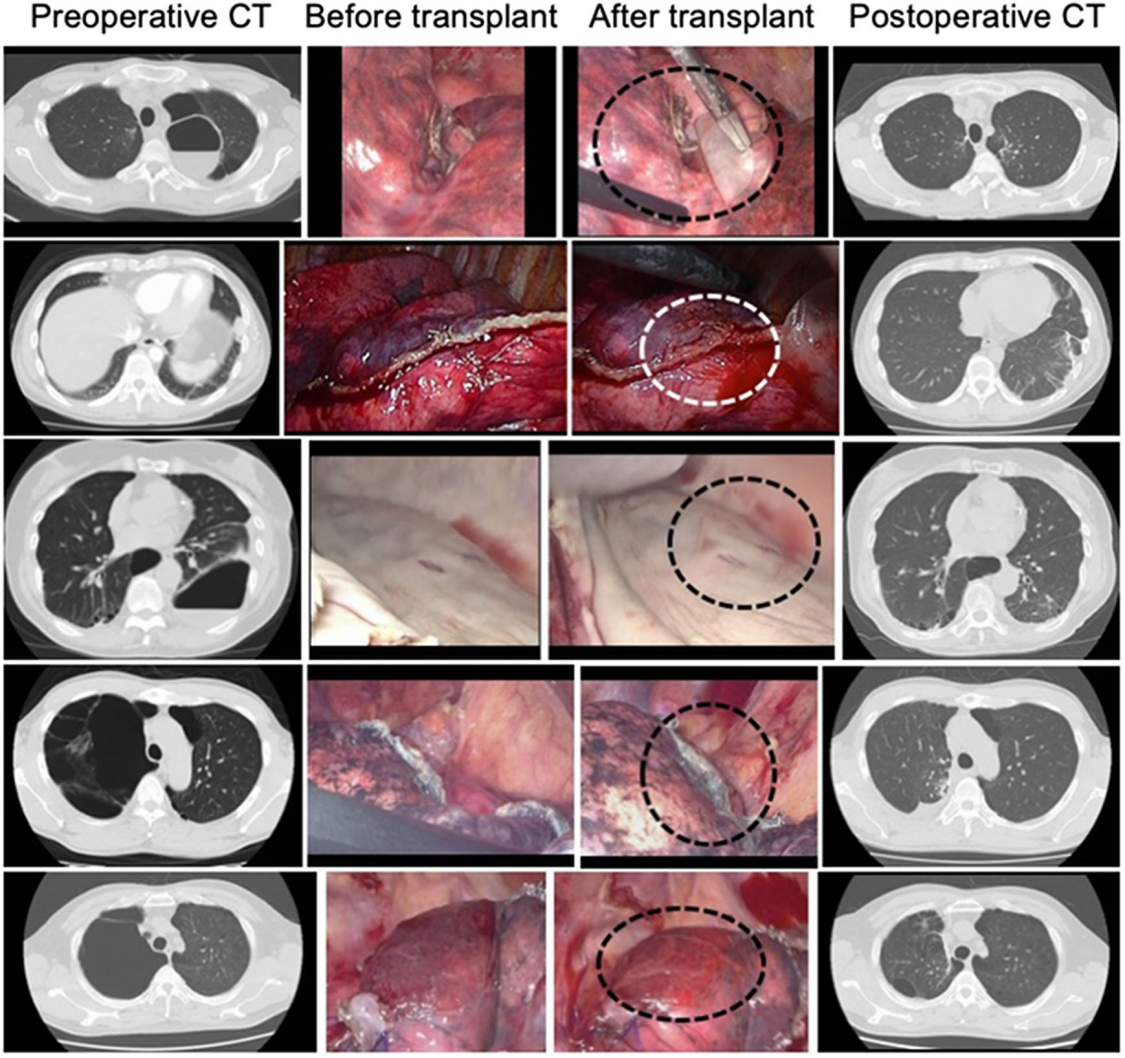
Chest computed tomography and intraoperative findings. Left, middle, and right columns of photographs represent preoperative chest computed tomography (CT), before fibroblast sheet transplantation from intraoperative digital video data, after fibroblast sheet transplantation from intraoperative digital video data, and postoperative chest CT, respectively. From case 1 to case 5 in this order from the upper row. The area of air leaks is surrounded by dashed lines in the middle column.

Surgical procedures involved thoracoscopic bullectomy in three patients and thoracoscopic lung wedge resection in two patients (Table 5). Both patients with thoracoscopic bullectomy had major LAL intraoperatively; the other three patients had minor LAL. Surgical time ranged from 184 to 292 min (mean 234.8 ± 36 min). Blood loss ranged from 2 to 80 mL (mean 27.0 ± 15 mL). The numbers of transplanted DFS to LAL sites ranged from 2 to 8 sheets (mean 6 ± 2 sheets). No patients required revision to thoracotomy, and 2 major LAL patients had air leaks persisting beyond 7 days (Fig. 3, “Before and After transplant”). Chest drainage duration ranged from 2 to 16 days (mean 5.0 ± 4.8 days). Chest tube re-insertion was not required, and no patients were followed by pleurodesis. The length of hospital stay ranged from 5 to 18 days (mean 8.8 ± 4 days). Patients had no adverse events, except one patient required hospital re-admission 2 months post-surgery for contralateral pneumonia.

**Table 5:** Clinical characteristics, operative characteristics, and outcomes.

Patient number	1	2	3	4	5
Age (years)	44	44	74	46	49
Sex	Man	Man	Woman	Man	Man
Diagnosis	Bullae	PAVM, TD	Bullae, COPD	Bullae	Bullae
Surgical procedure	Bullectomy	WR	Bullectomy	WR	Bullectomy
Air leakage	Minor	Minor	Major	Minor	Major
Op time (minutes)	246	196	256	184	292
Blood loss (mL)	2	35	7	11	80
Number of TCS	6	3	8	6	6
DD (days)	4	2	12	2	16
Hospital stay (days)	7	9	14	5	18

*PAVM* pulmonary arteriovenous malformation, *TD* Takayasu’s disease, *COPD* chronic obstructive pulmonary disease, *WR* wedge resection, *Op* operation, *TCS* transplanted cell sheet, *DD* drainage duration.

All patients have shown no LAL recurrence after discharge. All patients exhibited no abnormal findings in sheet-transplanted lungs by chest CT at 6 months post-surgery (Fig. 3, “Postoperative CT”).

## Discussion

LAL still represents a major problem in surgical lung resection. Advances in surgical techniques, including the use of stapling devices and tissue sealants such as fibrin glue, have minimized but not abolished such complications^8–11,15–19^. LAL after lung resections contributes to increased patient morbidity, delayed removal of chest tubes, and prolonged hospitalization. Attaar et al. have reported an incidence rate of prolonged lung air leak of 8.6%; patients with LALs after lung resection have significantly prolonged median lengths of hospital stays with higher rates of in-hospital mortality^6^. Pompili et al.^17^ analyzed the ESTS Database Annual Report and found that the incidence of PAL ( > 5 days) after lobectomy was still 9.9%^18^. LAL is not only associated with prolonged hospital stays but also may lead to more severe complications, such as emphysema, pneumonia, respiratory failure, and acute respiratory distress syndrome^8–11^. Despite developments and advances of surgical instruments including staplers, LAL remains among the most common adverse events after lung resection^6^.

Standard techniques are used to reduce all LAL followed by repeat lung submersion testing and analysis of leaks. Various surgical methods have sought to close or seal pleural defects and have been advocated to encourage healing and prevent fistulization, including pleural flap, dermal grafts, muscle bundles, omentum, pericardial flaps, biodegradable materials, and staple devices^15,16,18^. Notably, fibrin-based sealants have often been applied as pulmonary sealants due to their broad surgical experience and availability, high biocompatibility, and low toxicity^20–23^. However, fibrin sealants exhibit low tissue adhesive strength and have not been reliable or effective when routinely used in lung resection^19^.

As CT technology has advanced, CT has become the ultimate imaging technique for assessing patients who have pleuro-pulmonary diseases before surgery^24^. HRCT specifically provides useful patient anatomical information for surgeons^25^. Therefore, skilled surgeons can predict intraoperative LALs based on CT images^26,27^. By predicting intraoperative LAL occurrence using CT imaging, we have reported the development of a novel LAL sealant using tissue-engineered cell-based living sheets comprising patient-derived cells that spontaneously adhere to lung tissue surfaces^12–14,24,25^. Previously, we described the use of temperature-responsive culture dishes to fabricate transplantable cell sheets without using additional synthetic or biological materials or scaffolds. Under normal culture conditions at 37 °C, various cell types can attach, spread, and proliferate on these unique culture surfaces, similar to that on ordinary tissue culture dishes. However, by simply reducing culture temperature to 20 °C, cultured cells along with their deposited extracellular matrix (ECM) can be non-invasively harvested as intact sheets, without the need for destructive proteolytic enzymes. With non-destructive cell sheet harvest, cell-to-cell junctions and ECM proteins can therefore be maintained intact in the sheet construct^28–31^. During spontaneous temperature-triggered cell detachment processes, cell sheets contract under cytoskeletal reorganization, but can be re-expanded by applied external forces while maintaining their function and intact structures. These cell sheets can attach to tissue surfaces, using their natural adhesive proteins; DFS readily adhere to pleuro-pulmonary surfaces with slight applied pressure and are re-bondable. Cell sheets are also elastic by nature to adapt to local tissue mechanical dynamics as found in lung cyclic expansion and contraction during respiration^12^. For closing LALs in the pig model, an autologous DFS was transplanted directly to the pleural defect using a square-shaped support membrane. Within 5 min, the DFS produced stable attachment to the surrounding lung surface, without the use of sutures, fibrin glue, or staples. To create reinforced sealants, a second autologous DFS was transplanted directly over the first, and closure of the LALs caused by injury to the visceral pleura, was confirmed by the absence of air bubbles from the defect^13^.

We emphasize that the ideal tissue adhesive should have an outstanding safety profile with no health risks. Generally, the material should be safe, effective, reproducible, easily manipulated, and approvable by regulatory agencies (e.g., FDA). Autologous skin fibroblasts, therefore, provided several key advantages that are seemingly attractive for future clinical applications. Fibroblasts selected in this study are naturally involved in wound healing^12–14^. In the acute wounding inflammatory phase, local fibroblasts are attracted and activated by multiple cell-secreted growth factors and begin wound repair at 3–5 days after injury^12,13^. Migrating and proliferating fibroblasts secrete growth factors and matrix components. As DFSs do not require initial fibroblast recruitment, migration, and proliferation in the wound site, DFS applied to wounds can accelerate wound-healing processes.

Providing a strong and expandable framework for thin alveolar epithelial-capillary intersection, the pulmonary interstitial ECM contains elastic fibers responsible for tensile strength, elastic recoil, and tissue compliance. Fibroblasts are responsible for secreting a non-rigid ECM under normal conditions and during wound healing compatible with pulmonary ECM elasticity. Based on our previous results, we had evaluated the use of autologous dermal fibroblasts. Transplanted fibroblast sheets secreted FGF-2 and produced large amounts of ECM rich in type I and type III collagens^12–14,32^. Furthermore, α-SMA was expressed by transplanted DFSs at 4 weeks, indicating that the DFSs contained activated myofibroblasts. In this study, similar results were found in the harvested DFSs. Notably, harvested DFSs were revealed expression of α-SMA and contained activated myofibroblasts. Typically, during tissue repair after injury and surgery, fibroblasts including myofibroblasts migrate and proliferate^33,34^. When DFSs were transplanted after lung injury, deposition of abundant and diverse ECM enabled cell sheet sealants to respond mechanically and maintain stable LAL closure. Dermal fibroblasts possess high proliferative capabilities and are easily obtained from small, non-invasive biopsies for patient-specific ex vivo expansion under routine cell therapy conditions. While current culture methods used the fetal bovine serum, autologous serum could easily be used for future human cell expansion applications to eliminate xeno-derived media products. While human DFSs fabrication requires a 4-week cultivation period prior to surgery, in many countries including Japan, surgery patients are required to provide autologous blood for transfusion use prior to many (e.g., cardiothoracic) surgeries. As surgery is scheduled at least 2 weeks after sampling autologous blood, a 4-week biopsy/cell sheet cultivation period is within routine surgical schedule tolerance. Nonetheless, we have recognized that the clinical application of fresh DFS is limited by problems related to the necessary cell culture period, mass production schedule, preservation, and transportation logistics. In order to translate this technique more widely and expand DFS use to patients with acute surgical needs, HLA matching biobanks equipped with cryopreservation of DFSs should be considered in the future.

Many LALs resolve spontaneously within 48 h. Therefore, our results have not yet demonstrated that DFSs effectively act alone as intraoperative air leak sealants^11,17,19^. However, compared with conventional surgical methods for closure of LALs using applied biodegradable biomaterials with/without fibrin glue, the DFS clinical outcome was not inferior to chest tube duration (range, 2–14 days) and length of hospital stay data (3–17 days) during the same period. No recurrence of LAL was observed during the patients’ 6-month follow-up. The surgical use of autologous DFSs has been demonstrated to permanently close troublesome LALs using a readily available autologous cell source. Autologous DFSs are not only useful to close LALs but also have the potential to accelerate tissue repair. This approach might be expected to reduce surgical complications during high-risk lung resections.

## Methods

### Study design and setting

This study was designed as a single-arm, nonrandomized, uncontrolled study and was approved by the Human Ethics Committee of Tokyo Women’s Medical University and the Institutional Review Board for clinical research at Osaka University. This study was conducted according to the act for clinical research using human stem cells established by the Ministry of Health, Labor and Welfare of Japan and registered in the University Hospital Medical Information Network (UMIN) Clinical Trials Registry as No. UMIN000022554. Oral and written informed consent was obtained from all patients.

### Criteria

Patients who meet all inclusion criteria and do not meet any exclusion criteria shall be eligible.

1. Target diseases
  Pleural-pulmonary disease complicated with LAL during surgery: pneumothorax, bullae, and benign pulmonary tumors
  Reasons for inclusion: With advances in image processing and diagnostic imaging, preoperative chest CT has facilitated improved examination of emphysematous changes, i.e., prediction of pleural-pulmonary disease that may cause LAL during surgery. Therefore, at present, we consider that a cultured autologous DFS can be transplanted for the above diseases. In addition, these diseases will also be examined in subsequent clinical research for effectiveness by assessing safety in each case.
2. Inclusion criteria
  (i) 20 years or older at the time of informed consent acquisition;
  (ii) Surgery involving lung resection and/or pulmonary-pleural resection;
  (iii) Removal of autologous skin tissue without functional invasion
  (iv) Functional preservation of the main organs, such as bone marrow, liver, and kidneys, with the following criteria, met within 2 weeks before registration:
    1. White blood cell count: ≥3000–≤12,000/mm^3^;
    2. Neutrophil count: ≥1500–≤5000/mm^3^;
    3. Hemoglobin: ≥8 g/dL;
    4. Platelet count: ≥50,000/mm^3^;
    5. AST and ALT: ≤2.5 times the upper limits of the institutional normal values; and
    6. Serum creatinine: ≤1.5 times the upper limit of the institutional normal value;
  (v) Patients who gave consent voluntarily after receiving sufficient explanation before participating in the research.
3. Exclusion criteria
  (i) Positivity for infectious diseases (HBV, HCV, HIV, HTLV, and syphilis);
  (ii) Conditions precluding the closure of pulmonary air leakage;
  (iii) Pregnancy or suspected pregnancy;
  (iv) Allergy to lidocaine hydrochloride or antibiotics (gentamicin and fungizone);
  (v) Psychosis or psychiatric symptoms (depression, mania, delirium, hallucinations, and dementia) precluding participation in the research;
  (vi) Treatment or hospital visit for systemic skin disorders, determined to be inappropriate by attending physicians; and
  (vii) Others determined to be inappropriate by attending physicians.

### Patient selection

All eligible patients met all inclusion criteria and did not meet any exclusion criteria. All patients were examined by thin-sliced (1-mm images) high-resolution CT (HRCT) in the absence of intravascular contrast material. With the patient in the supine position, HRCT scans were obtained during full inspiration. The first patient was enrolled in May 2016 and the last in March 2018, five patients had intraoperative air leaks during thoracoscopic lung resection. These patients consisted of four men and one woman, ranging in age from 44 to 74 years (mean: 51.4 ± 12.8 years) (Table 5). Clinical diagnoses were a bullous disease in four patients and a benign lung tumor (pulmonary arteriovenous malformation) in one patient (Fig. 3, Preoperative CT). Of five patients, four patients obtained emphysematous changes by HRCT and the remaining one patient with the benign lung tumor had been taken a long-term steroid therapy for Takayasu’s disease.

LALs were classified into minor and major leaks. LAL pleural defects of more than 3 square centimeters were defined as major leaks; smaller were defined as minor leaks.

### Preparation of autologous serum

Blood was peri-operatively collected using a 30-mL syringe and a safety-type winged 21-G needle in the operation room. Blood volume was ~100 mL collected into 50-mL labeled (identification number and sampling date) centrifuge tubes. These samples were stored at room temperature and used to prepare sera within 1 day after sampling. The serum was separated from the collected blood. Crude serum was collected twice by centrifugation at 3100 rpm for 10 min and sterilized by liquid filter sterilization in a biosafety cabinet. Subsequently, filtered serum was dispensed into a 15-mL labeled (identification number, batch number, and expiration date) centrifuge tube and stored at −30 °C.

### Fabrication of autologous dermal fibroblast sheets

Patients were placed in the lateral decubitus position and with the eventual surgical access site facing upwards. An ~1-cm^2^ skin specimen was excised from the anticipated surgical incision site under local anesthesia (Fig. 1A). The skin specimen was transferred into a 50-mL centrifuge tube with Dulbecco’s modified Eagle’s medium (DMEM, Merck, Darmstadt, Germany) containing 57 µg/mL ampicillin sodium and 28 µg/mL sulbactam sodium (Unasin-S, Pfizer, NYC, USA), 99 µg/mL streptomycin sulfate (Meiji Seika Pharma, Tokyo, Japan), and 0.99 µg/mL amphotericin B (Fungizone, Bristol-Myers Squibb, NYC, USA), and transported to biosafety cabinet in the clean room of the hospital cell processing facility.

Approximately 3 mm^2^ of tissue was prepared using surgical scissors and forceps. Excised skin tissues were immersed in DMEM containing 1000 U/mL dispase I (Godo Shusei, Tokyo, Japan), 57 µg/mL ampicillin sodium 28 µg/mL sulbactam sodium, 99 µg/mL streptomycin sulfate, and 0.99 µg/mL amphotericin B in 35-mm culture dish, followed by enzymatic treatment in carbon dioxide gas cell incubator for 2–3 h (Fig. 1B). For fibroblast isolation, enzyme-treated tissues were transferred into a 35-mm dish with DMEM containing 57 µg/mL ampicillin sodium 28 µg/mL sulbactam sodium, 99 µg/mL streptomycin sulfate, and 0.99 µg/mL amphotericin B and washed by shaking. The epidermis was held with one forceps while the dermis was held with another forceps, and the epidermis removed by peeling (Fig. 1C). Peeled epidermal tissue pieces were collected in a cryogenic vial for tissue cryopreservation. Adipose tissue was physically removed as much as possible; then, dermal tissue was transferred into a new 60-mm dish and cut into ~1-mm square pieces using surgical scissors and forceps. These tissue pieces were spread over the dish (Fig. 1D), and the lid was left open and allowed to dry in a biosafety cabinet until tissue pieces would not move even when touched with forceps. Approximately 4 mL DMEM containing 10% autologous serum and antibiotics consisting in 40 µg/mL gentamicin sulfate (Gentacin, Takata Pharmaceutical, Saitama, Japan), and 0.5 µg/mL amphotericin B (Fungizone) was added carefully to the 60 mm dishes, followed by culture in a carbon dioxide cell culture incubator. On day 6 of explant culture, first observation and replacement of culture medium were performed. Total days of explant culture are shown in Table 1.

For fibroblast subculture, cultured cell morphology was first observed under a microscope, then the medium was removed, followed by washing with 2 mL of Dulbecco’s phosphate-buffered saline (PBS) in a biosafety cabinet. The cells were treated with 0.5 mL trypsin-ethylenediamine tetraacetic acid (EDTA), and culture dishes were placed in a carbon dioxide incubator for 3–5 min. Subsequently, 1.5 mL of DMEM containing 10% autologous serum, 40 µg/mL gentamicin sulfate, and 0.5 µg/mL amphotericin B was added to the dishes in a biosafety cabinet to inhibit further trypsin reaction. The cells suspended in DMEM were transferred into a 50-mL centrifuge tube and centrifuged at 1000 rpm for 5 min at 4 °C, and the supernatant was removed. The number of cells was counted using 20 μL each of the cell suspension and trypan blue on a hemocytometer. The cells were then seeded at a density of 3–5 × 10^3^ cells/cm^2^ in as many dishes as possible.

After 4 or 5 days of the subculture, these fibroblasts were also treated with trypsin-EDTA for collecting and seeding onto 60-mm diameter temperature-responsive culture dish (UpCell^TM^, CellSeed, Tokyo, Japan) at a density of 1 × 10^4^ cells/cm^2^ to fabricate autologous dermal fibroblast cell sheets in DMEM containing 10% autologous serum, 200 µM L-ascorbic acid phosphate magnesium salt *n*-hydrate (Wako pure chemical, Osaka, Japan), 40 µg/mL gentamicin sulfate, and 0.5 µg/mL amphotericin B. After 3 days of cell culture on temperature-responsive culture dishes, cells became confluent, and the culture medium was replaced new culture medium at 3, 6, 9, and 10 days after the subculture onto temperature-responsive culture dishes. After 10 days of cultivation, intact 30 mm diameter patient-specific dermal fibroblast sheets (DFS) were ready for harvest using support membranes (CellShifter^TM^, CellSeed, Tokyo, Japan).

### Quality control tests of cell sheets

Before transplantation of autologous DFS, quality control tests were performed for confirming DFS quality and purity. Culture supernatants of explant cultures of dermal tissues and DFS cells on temperature-responsive culture dishes were confirmed for sterility by microbial culture to detect aerobic and anaerobic bacteria and fungi, loop-mediated isothermal amplification (LAMP) to detect DNA derived from *Mycoplasma pneumoniae*, and limulus amebocyte lysate assay to detect endotoxin. Autologous dermal fibroblasts cultured on temperature-responsive culture dishes for 9 days were also subject to quality control tests. Cultured fibroblasts as a DFS (cell sheet) were harvested by reducing culture temperature to 20 °C and observed by optical microscopy to confirm that the cell sheet completely contained cultured fibroblasts and that the fibroblast sheet was defect-free. Fibroblast sheets without defects were treated with trypsin-EDTA to prepare cell suspensions to assess total cell numbers and cell viability by trypan blue dye exclusion assay. These DFS cells in suspensions were also subject to flow cytometry to determine vimentin (mouse monoclonal anti-vimentin; V9, sc-6260 FITC, Santa Cruz Biotechnology, CA, USA; dilution 1:100)-positive cells to assert fibroblast purity in the harvested sheet.

### Transplantation of tissue-engineered autologous DFS to intraoperative LAL

The cultured autologous DFS were harvested from temperature-responsive dishes in a biosafety cabinet placed in clean room of cell processing facility and washed with Hanks’ balanced salt solution (HBSS) to remove the culture medium. CellShifters were also washed with HBSS and placed onto the DFS. Culture dishes containing the DFS covered with CellShifters were put into autoclaved transportation containers in the biosafety cabinet, and these containers were put in a temperature-stable bag in the facility to transport the DFS to the surgery room for transplantation. Subsequently, the cultured DFS were recovered from UpCell culture dishes at room temperature using CellShifters as a support and transport/handling membrane in parallel with the surgery.

After lung resection, LAL was confirmed by the continuous appearance of air bubbles upon lung submergence in physiological saline. As LAL from the surgically operated lung was recognized, DFSs were applied from the CellShifter directly to each LAL site on the surgically operated lung in a collapsed state under one-lung ventilation and held to the tissue surface under slight pressure. Five minutes later, the support membrane was removed from the first DFS. Similarly, an additional cultured DFS was layered over the initial DFS first fixed to the LAL site and the support membrane removed 5 min later. Subsequently, bilateral ventilation was performed to check for LAL at an airway pressure of 15 cm H_2_O. DFSs were transplanted repeatedly until no LAL could be observed. After DFS transplantation, bilateral ventilation was performed again to check the LAL at an airway pressure of 15 cm H_2_O. As no LAL was evident with this check, surgery was then completed following standards of care. After DFS transplantation surgery, LALs were managed by a digital drainage system. Routine examinations were conducted to blood chemistry test and chest roentgen at 1, 3, 5, and 7 postoperative days (POD). Chest CT was performed at 7 POD and 1 month, 3 months, and 6 months after surgery. Each patient was monitored for 6 months.

### Histological and immunohistochemical analyses

DFSs were fixed with 10% formalin and routinely processed into 10-µm-thick paraffin-embedded sections. Hematoxylin and eosin (HE), Azan staining, and immunohistological staining were performed by conventional methods. In the immunohistological analysis, the DFS were treated with one of the following primary antibodies; mouse monoclonal anti-vimentin (V9, NCL-VIM-V9, Leica Microsystems, Wetzlar, Germany; dilution 1:500), mouse monoclonal anti-alpha-smooth muscle actin (α-SMA, 1A4, M0815, Dako, Agilent Technologies, CA, USA; dilution 1:1000), rabbit polyclonal anti-collagen type 1 (ab34710, Abcam, Cambridge, UK; dilution 1:1000), goat polyclonal anti-collagen type 3 (1330-01, Southern Biotechnology Associates, AL, USA; dilution 1:200), and rabbit polyclonal anti-fibroblast growth factor 2 (FGF-2, sc-79, Santa Cruz Biotechnology, CA, USA; dilution 1:200).

### Statistical analysis

Descriptive statistics of continuous variables are expressed as mean, standard deviation, and minimum and maximum values, while categorical variables were expressed as number and percentage.

### Reporting summary

Further information on research design is available in the Nature Research Reporting Summary linked to this article.

## Supplementary information

Reporting Summary Checklist

## Data Availability

The data that support the findings of this study are available from the corresponding author upon reasonable request.
