# Supplementary material for: Bio-artificial pleura using autologous dermal fibroblast sheets to mitigate air leaks during thoracoscopic lung resection
Source: NPJ Regen Med. 2021 Jan 4;6:2. doi: 10.1038/s41536-020-00113-z (PMC7782673; doi:10.1038/s41536-020-00113-z)
Supplement: Supplementary file 1 — Reporting Summary Checklist [file 41536_2020_113_MOESM1_ESM.pdf]

nature research

Corresponding author(s): NPJREGENMED-00345R2
Masato Kanzaki

Last updated by author(s): Nov 17, 2020

Reporting Summary

Nature Research wishes to improve the reproducibility of the work that we publish. This form provides structure for consistency and transparency in reporting. For further information on Nature Research policies, see our Editorial Policies and the Editorial Policy Checklist.

Statistics

For all statistical analyses, confirm that the following items are present in the figure legend, table legend, main text, or Methods section.

n/a	Confirmed	
☐	☒	The exact sample size (*n*) for each experimental group/condition, given as a discrete number and unit of measurement
☐	☒	A statement on whether measurements were taken from distinct samples or whether the same sample was measured repeatedly
☐	☐	The statistical test(s) used AND whether they are one- or two-sided<br>*Only common tests should be described solely by name; describe more complex techniques in the Methods section.*
☐	☐	A description of all covariates tested
☐	☐	A description of any assumptions or corrections, such as tests of normality and adjustment for multiple comparisons
☐	☐	A full description of the statistical parameters including central tendency (e.g. means) or other basic estimates (e.g. regression coefficient) AND variation (e.g. standard deviation) or associated estimates of uncertainty (e.g. confidence intervals)
☐	☐	For null hypothesis testing, the test statistic (e.g. *F*, *t*, *r*) with confidence intervals, effect sizes, degrees of freedom and *P* value noted<br>*Give P values as exact values whenever suitable.*
☐	☐	For Bayesian analysis, information on the choice of priors and Markov chain Monte Carlo settings
☐	☐	For hierarchical and complex designs, identification of the appropriate level for tests and full reporting of outcomes
☐	☐	Estimates of effect sizes (e.g. Cohen's *d*, Pearson's *r*), indicating how they were calculated

Our web collection on statistics for biologists contains articles on many of the points above.

Software and code

Policy information about availability of computer code

Data collection	There is no specific software to collect the data in this single-arm study.
Data analysis	There is no specific software to analize the data in this single-arm study.

For manuscripts utilizing custom algorithms or software that are central to the research but not yet described in published literature, software must be made available to editors and reviewers. We strongly encourage code deposition in a community repository (e.g. GitHub). See the Nature Research guidelines for submitting code & software for further information.

Data

Policy information about availability of data

All manuscripts must include a data availability statement. This statement should provide the following information, where applicable:

- Accession codes, unique identifiers, or web links for publicly available datasets
- A list of figures that have associated raw data
- A description of any restrictions on data availability

The data that support the findings of this study are available from the corresponding author upon reasonable request.

Field-specific reporting

Please select the one below that is the best fit for your research. If you are not sure, read the appropriate sections before making your selection.

☒ Life sciences ☐ Behavioural & social sciences ☐ Ecological, evolutionary & environmental sciences

For a reference copy of the document with all sections, see nature.com/documents/nr-reporting-summary-flat.pdf

Life sciences study design

All studies must disclose on these points even when the disclosure is negative.

Sample size	Five
Data exclusions	No exclusions
Replication	The reproducibility of safety of transplantation of fibroblast sheet was confrmed by evaluating in this study.
Randomization	There is no randomization.
Blinding	There is no blinding.

Reporting for specific materials, systems and methods

We require information from authors about some types of materials, experimental systems and methods used in many studies. Here, indicate whether each material, system or method listed is relevant to your study. If you are not sure if a list item applies to your research, read the appropriate section before selecting a response.

Materials & experimental systems

n/a	Involved in the study	
☐	☒	Antibodies
☐	☒	Eukaryotic cell lines
☒	☐	Palaeontology and archaeology
☒	☐	Animals and other organisms
☒	☐	Human research participants
☐	☒	Clinical data
☒	☐	Dual use research of concern

Methods

n/a	Involved in the study	
☒	☐	ChIP-seq
☐	☒	Flow cytometry
☒	☐	MRI-based neuroimaging

Antibodies

Antibodies used

Antibodies used for immunohistological analysis
1. Anti-vimentin mouse monoclonal antibody. Clone: V9. Leica Microsystems, Cat. No. NCL-LVIM-V9.
2. Anti-alpha-smooth muscle actin. Clone: 1A4. Dako, Cat. No. M0851.
3. Anti-collegan type 1 rabbit polyclonal antibody. abcam, Cat. No. ab34710.
4. Anti-collagen type 3 goat polyclonal antibody. Southern Biotechnology Associates, Cat. No. 1330-01.
5. Anti-fibroblast growth factor 2 rabbit polyclonal antibody. Santa Cruz, Cat. No. sc-79.

Antibodies used for Flow cytometry
Anti-vimentin mouse monoclonal antibody. Clone: V9. Santa Cruz, Cat. No. sc-6260.

Validation

Human positive control tissue slides were used for validation of the immunohistological analysis. Human dermal fibroblasts derived from 3 volunteer donors were used for validation of the flow cytometry.

Eukaryotic cell lines

Policy information about cell lines

Cell line source(s)	Autologous human dermal fibroblasts were derived from skin tissues from 5 patients.
Authentication	Preparation procedures were carried out in accordance with GMP guideline and the results were recorded.

Mycoplasma contamination	No contaminations with Mycoplasma were confirmed by LAMP method and culture test of Mycoplasma.
Commonly misidentified lines (See ICLAC register)	Commonly misidentified line was not used.

Clinical data

Policy information about clinical studies

All manuscripts should comply with the ICMJE guidelines for publication of clinical research and a completed CONSORT checklist must be included with all submissions.

Clinical trial registration	This study was registered in the University Hospital Medical Information Network (UMIN) Clinical Trials Registry as No. UMIN000022554.
Study protocol	The full trial protocol which registered in the University Hospital Medical Information Network (UMIN) Clinical Trials Registry as No. UMIN000022554 can be accessed. Further more, the full trial protocol, translated into English, was uploaded as a supplement.
Data collection	From May 2016 to December 2018
Outcomes	Primary endpoints<br>Safety of the cell sheet is evaluated.<br>- Recurrence of pulmonary air leakage due to dropping off or breakage of the cell sheet;<br>- Infection caused by cell sheet transplantation; and<br>- Safety of cell sheet transplantation procedures.<br><br>Secondary endpoints<br>Evaluation of pulmonary air leakage (thoracic cavity drainage and hospitalization periods)

Flow Cytometry

Plots

Confirm that:

☒ The axis labels state the marker and fluorochrome used (e.g. CD4-FITC).

☒ The axis scales are clearly visible. Include numbers along axes only for bottom left plot of group (a 'group' is an analysis of identical markers).

☐ All plots are contour plots with outliers or pseudocolor plots.

☒ A numerical value for number of cells or percentage (with statistics) is provided.

Methodology

Sample preparation	BD Cytofix/Cytoperm Fixation/Permeabilization Kit (BD Bioscience, Cat. No. 554712) was used for sample preparation.
Instrument	Gallios (Beckman Coulter).
Software	Gallious software (ver.1.2, Beckman Coulter) was used for collecting data, and Kaluza (ver 1.1, Beckman Coulter) was used for analysis of the data.
Cell population abundance	Before starting this clinical study, we confirmed that cell population consisted dermal fibroblast by using fibroblast derived from skin tissues of volunteer donors. Procedures of fibroblast preparation and quality control test of this clinical study were carried out in accordance with the results of the pre-clinical study.
Gating strategy	Isotype control of anti-vimentin antibody was used. The gate indicating vimentin-positive cells does not include cells treated with the control antibody (<1%).

☐ Tick this box to confirm that a figure exemplifying the gating strategy is provided in the Supplementary Information.
